# Characteristics of the complete chloroplast genome sequences of *Stylidium debile* and *Stylidium petiolare* (Stylidiaceae)

**DOI:** 10.1080/23802359.2021.1985403

**Published:** 2021-10-07

**Authors:** Lin Li, Guo-Ming Liu, Zhi-Rong Zhang, Richard T. Corlett, Wen-Bin Yu

**Affiliations:** a Center for Integrative Conservation, Xishuangbanna Tropical Botanical Garden, Chinese Academy of Sciences, Menglun, China; b Center of Conservation Biology, Core Botanical Gardens, Chinese Academy of Sciences, Menglun, China; c Chinese Carnivorous Plants Garden, Tongxiang, China; d Germplasm Bank of Wild Species, Kunming Institute of Botany, Chinese Academy of Sciences, Kunming, China; e Southeast Asia Biodiversity Research Institute, Chinese Academy of Science, Nay Pyi Taw, Myanmar

**Keywords:** Chloroplast genome, plastome, *Stylidium debile*, *Stylidium petiolare*, Stylidiaceae

## Abstract

We report complete chloroplast genome (plastome) sequences of *Stylidium debile* (150,105 bp) and *Stylidium petiolare* (150,998 bp). Both plastomes had the typical quadripartite structure, with large single-copy (LSC) and small single-copy (SSC) regions separated by two inverted repeat (IR) regions. Both plastomes have lost the *rps19* and *ycf15* CDS genes, and had *infA-*like, *rps22-*like, and *rps7-*like pseudogenes. Moreover, IR regions were expanded by having *trnH*^GUG^ tRNA and the *rps22*-like pseudogene. Plastome phylogenomic analyses showed that the two *Stylidium* species formed a monophyletic clade (BS = 100), sister to the Argophyllaceae (BS = 86/83). Sequence differences between the two *Stylidium* plastomes were 5011 sites, including 2166 variable sites and 2845 indels, with the *petA-psbJ* spacer the most variable region, followed by the *trnK*^UUU^-*matK* intron and *trnG*^UUG^-*rps16* spacer.

*Stylidium* (Stylidiaceae) are herbaceous perennials with the vernacular name of trigger-plants because of their pollination mechanism (Carolin 2007). There are around 220 species, mostly endemic to Australia, but with a few species in Southeast Asia and Sri Lanka, and also in New Zealand and southern South America (Carolin 2007). Some species are considered to be protocarnivorous (or subcarnivorous) because the glandular hairs trap insects (Darnowski et al. 2006). To date, phylogenetic relationships among the Stylidiaceae and within *Stylidium* are not well resolved (Laurent et al. 1998; Wagstaff and Juliet 2002), and only a few DNA regions from several species of *Stylidium* are deposited in GenBank, with no chloroplast genome sequences (accessed on 9 May 2021). In this study, we therefore sequenced and analyzed the complete chloroplast genome (or plastome) sequences of two Australian species, *Stylidium debile* F. Muell. 1859 and *Stylidium petiolare* Sond. 1845, which can be used to develop highly variable and informative plastid DNA regions for phylogenetic and population genetic studies of Stylidiaceae and *Stylidium*.

Genomic DNA of *Stylidium debile* and *S. petiolare* was extracted by a modified CTAB method (Doyle and Doyle 1987) from the silica-gel dried leaves, which were collected from cultivated living plants in the greenhouse of the Chinese Carnivorous Plants Garden (Chongfu Town, Tongxiang, Zhenjiang, China; 30°30′55.5″N, 120°24′46.5′E). The genomic DNA samples were then purified and fragmented into the size of 350 bp for preparing the sequencing library. The library preparation followed Zeng et al. (2018). Around 2.0 GB clean data for each species were generated by Illumina Hi-Seq 2500 System via the 150 bp pair-end reads approach. Complete plastomes of these two species were *de novo* assembled by the GetOrganelle toolkit (Jin et al. 2020). The two plastome sequences were initially annotated by the GeSeq online tool (Tillich et al. 2017), then the start and stop codons were manually checked and adjusted by Geneious (Kearse et al. 2012). The voucher specimens (*Stylidium debile*: *Liu G.-M. QT05*; *Stylidium petiolare*: *Liu G.-M. QT22*) and DNA (*Stylidium debile*: *Yu W.-B. P476*; *Stylidium petiolare*: *Yu W.-B. P475*) are preserved in the herbarium of Xishuangbanna Tropical Botanical Garden, Chinese Academy of Sciences (HITBC, http://hitbc.xtbg.ac.cn/, Jian-Wu Li, ljw@xtbg.org.cn) and in the Germplasm Bank of Wild Species, Kunming Institute of Botany, Chinese Academy of Sciences (http://www.genobank.org/, Jun-Bo Yang, jbyang@mail.kib.ac.cn), respectively.

The two plastome sequences had a typical quadripartite structure, with two inverted repeat (IR) regions separating large single-copy (LSC) and small single-copy (SSC) regions. The plastome sizes of *Stylidium debile* (accession number: MZ239203) and *Stylidium petiolare* (accession number: MZ239204) were 150,105 bp (GC content: 37.9%; LSC: 81,752 bp; SSC: 18,341 bp; IR: 25,006 bp) and 150,998 bp (GC content: 30.8%; LSC: 81,869 bp; SSC: 18,285 bp; IR: 25,422 bp), respectively. Both plastomes contained 131 genes in total, including 80 protein coding (CDS) genes, 38 tRNAs, eight rRNAs, and four pseudogenes (*infA-*like and *rps22-*like in LSC, and *rps7-*like in IR). The IR region had 19 genes, including six CDS genes, eight tRNAs, and four rRNAs, *rps7-*like pseudogene, and partial *rps22*-like pseudogene. Compared with the plastome of *Amborella trichopoda* Baill. (AJ506156), these two *Stylidium* plastomes had lost *rps19* and *ycf15* CDS genes, and their IR regions were expanded by having *trnH*^GUG^ tRNA and an *rps22*-like pseudogene.

Twenty realigned plastomes of Asterales and one of *Ilex dumosa* Reissek (Aquifoliales, KP016927) as an outgroup with one IR region were aligned using MAFFT (Katoh and Standley 2013). Gaps in the initial matrix were removed by trimAl (Capella-Gutiérrez et al. 2009) using a command ‘-gt 0.9 -cons 60’. To infer the phylogeny of the Asterales, we performed maximum-likelihood analyses using RAxML (Stamatakis et al. 2008) by the GTR + GAMMA + I model with 1000 bootstraps for calculating support values for nodes/branches. The initial and trimmed matrixes were analyzed separately. Plastome phylogenies recovered the expected familial relationships among the Asterales (Stevens 2001; Li et al. 2019), with the exception that Argophyllaceae was sister to Stylidiaceae with moderate support values by initial (BS = 86) and trimmed datasets (BS = 83, see Figure 1). Moreover, *Stylidium debile* and *Stylidium petiolare* formed a monophyletic clade (BS = 100). There were 5011 sites with sequence differences, 2166 variable sites, and 2845 indels, between *Stylidium debile* and *Stylidium petiolare*. The *petA-psbJ* spacer was the most variable region, followed by the *trnK*^UUU^-*matK* intron (pi = 0.040–0.070) and the *trnG*^UUG^-*rps16* spacer (pi = 0.037–0.070). This study provides important plastomic information and identifies hypervariable regions that can be used in future studies on the phylogeny and evolution of the genus *Stylidium*.

**Figure 1.**
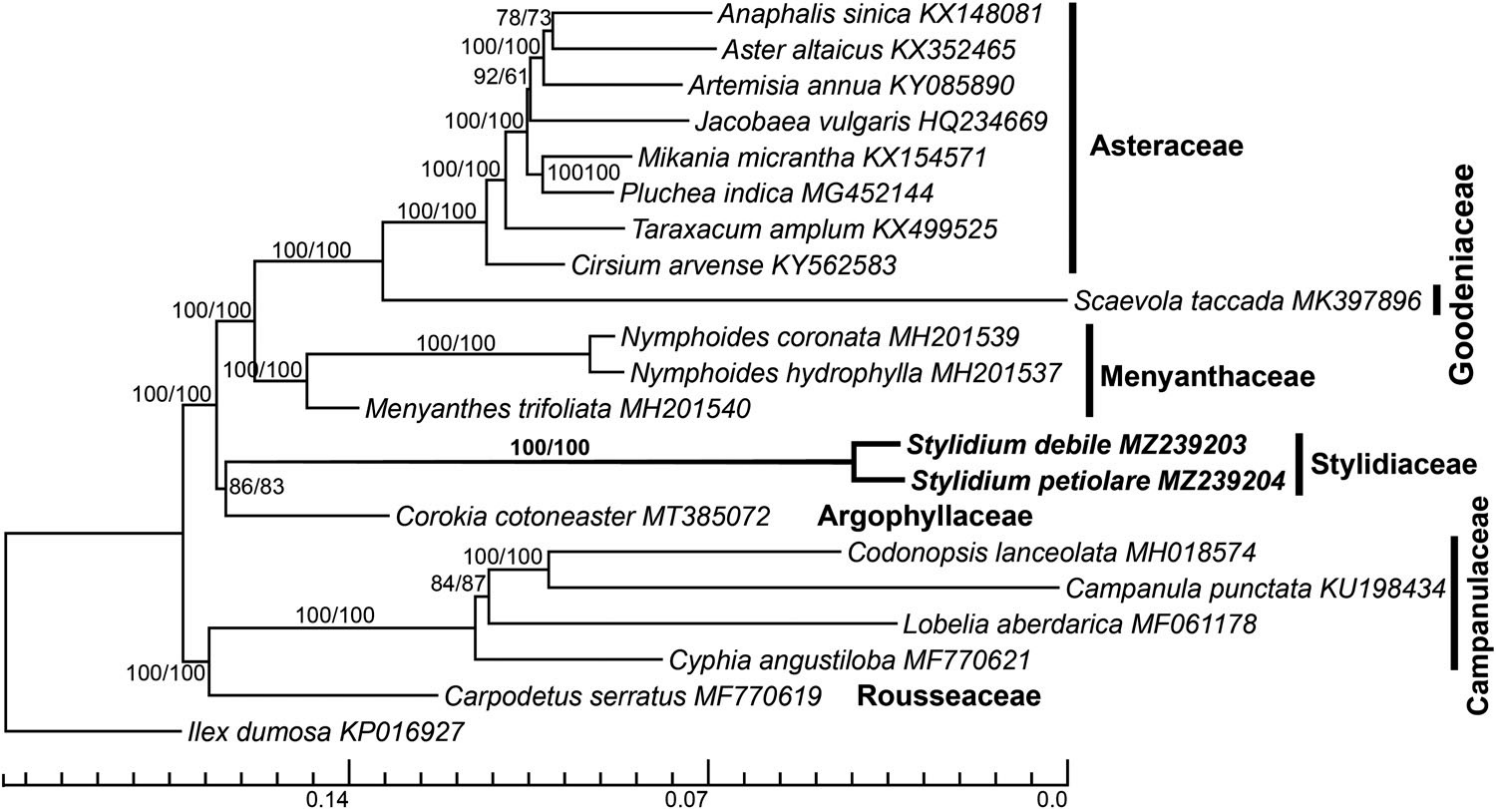
Plastome phylogenomics of Asterales inferred using the maximum-likelihood (ML) method. *Stylidium debile* and *Stylidium petiolare* are highlighted in bold. ML bootstrap values of nodes inferred using initial and trimmed matrixes, respectively, are presented next to the nodes. The bottom scale bar represents the number of substitutions per site.

## Data Availability

Two complete plastome sequences were deposited in GenBank with accession numbers MZ239203 and MZ239204, and are also available at Figshare (doi:10.6084/m9.figshare.14649921). The associated BioProject, SRA, and Bio-Sample numbers are PRJNA739677, SRR14871484–SRR14871485, and SAMN19789662–SAMN19789663, respectively. The data were collected without violation of the protection of human subjects, or other valid ethical, privacy, or security concerns.
